# Supplementary material for: Characterization of the pig lower respiratory tract antibiotic resistome
Source: Nat Commun. 2023 Aug 12;14:4868. doi: 10.1038/s41467-023-40587-1 (PMC10423206; doi:10.1038/s41467-023-40587-1)
Supplement: Supplementary file 1 — Supplementary information [file 41467_2023_40587_MOESM1_ESM.pdf]

Supplementary information for

Characterization of the pig lower respiratory tract antibiotic resistome

Yunyan Zhou[1,2,3], Jingquan Li[1,3], Fei Huang[1,3], Huashui Ai[1], Jun Gao[1], Congying Chen[1,*], Lusheng Huang[1,*]

[1] *National Key Laboratory of Swine Genetic Improvement and Germplasm Innovation, Jiangxi Agricultural University, Nanchang 330045, China*

[2]*Institute of Engineering Biology and Health, Collaborative Innovation Center of Yangtze River Delta Region Green Pharmaceuticals, College of Pharmaceutical Sciences, Zhejiang University of Technology, Hangzhou 310014, China*

[3]These authors contributed equally

[*]Correspondence authors: Lushenghuang@hotmail.com (Lusheng Huang), chcy75@hotmail.com (Congying Chen)

Running title: Antibiotic resistomes of pig lower respiratory tract microbiome

Supplementary Figures

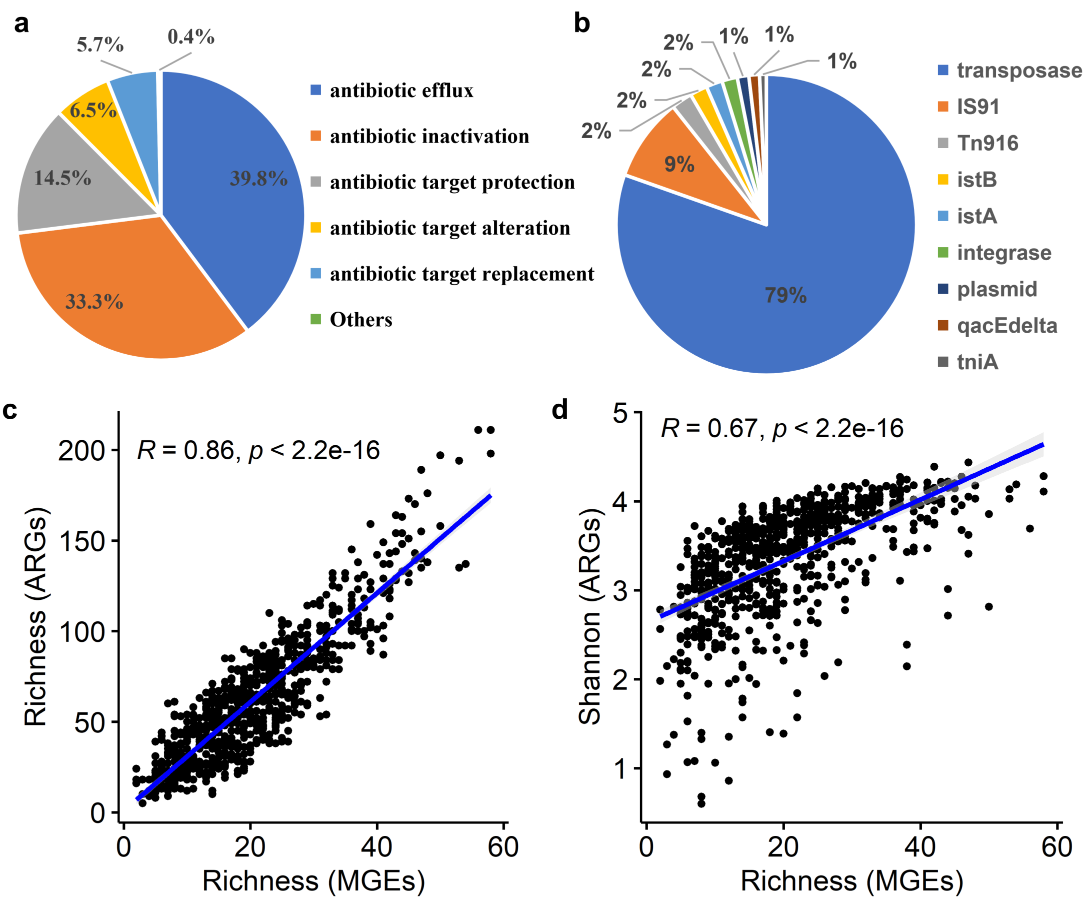

Supplementary Fig. 1. The proportion of each type of resistance mechanisms and MGEs, and the relationship of ARGs with MGEs

(a) The proportion of each type of resistance mechanisms based on the average abundance in 745 tested samples. (b) The proportion of each type of MGEs based on the average abundance in 745 tested samples. (c) The relationship between the richness (number) of MGEs and the richness of ARGs (number). (d) The relationship between the richness (number) of MGEs and the evenness (Shannon index) of ARGs. The correlation analyses were performed in bronchoalveolar lavage fluid samples from F_7 pigs of a mosaic population (n = 613). The two-sided Spearman rank correlation analyses were performed by the *ggscatter* function in the *ggpubr* R package. Data are presented as the actual value of the corresponding variable obtained for each sample (each point). Source data are provided as a Source Data file.

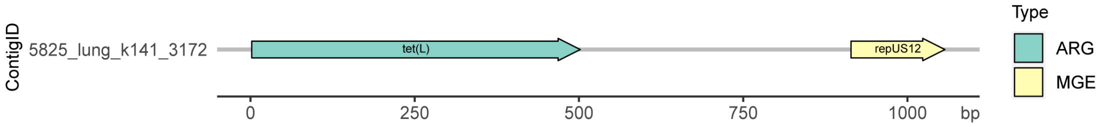

Supplementary Fig. 2. The close linkage relationship between *tet(L)* and plasmid *repUS12* in the contigs of pig lung microbiome. The green box represents the ARG *tet(L)* and the yellow box shows the MGE *repUS12*. Source data are provided as a Source Data file.

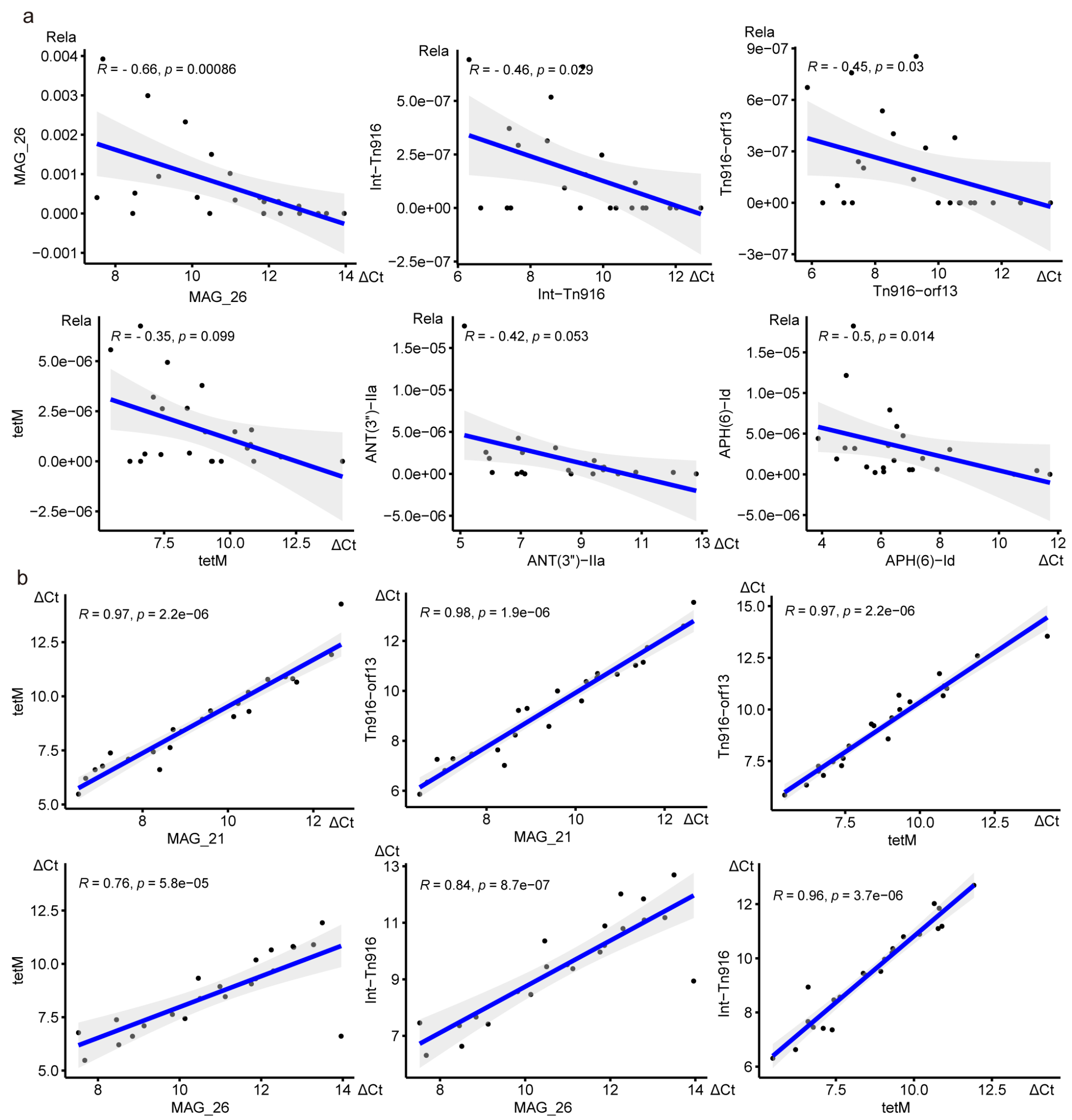

Supplementary Fig. 3. Confirmation of the abundance changes of ARGs, MGEs, and MAGs, and the co-abundance relationships between ARGs, MGEs and MAGs, and between ARGs and MGEs based on qPCR

(a) Confirmation of the abundance changes of ARGs, MGEs, and MAGs in tested samples (n = 23) by analyzing the correlations between the abundance values from qPCR (ΔCt) and the abundances from metagenomic sequencing. Because the MAG_340 was only detected in three of 23 samples in the metagenomic sequencing, the correlation analysis could not be performed although it was validated in the qPCR. However, MAG21 was not validated. (b) The co-abundance relationships between ARGs, MGEs, and MAGs carrying these genes, and between ARGs and MGEs. The co-abundance relationships were well validated for all pairs. The *x*-axis shows the abundance values by qPCR (ΔCt), and the *y*-axis represents the abundance values from metagenomic sequencing. The two-sided Spearman rank correlation analyses were performed by the *ggscatter* function in *ggpubr* R package. Data are presented as the actual value of the corresponding variable obtained for each sample (each point). Source data are provided as a Source Data file.

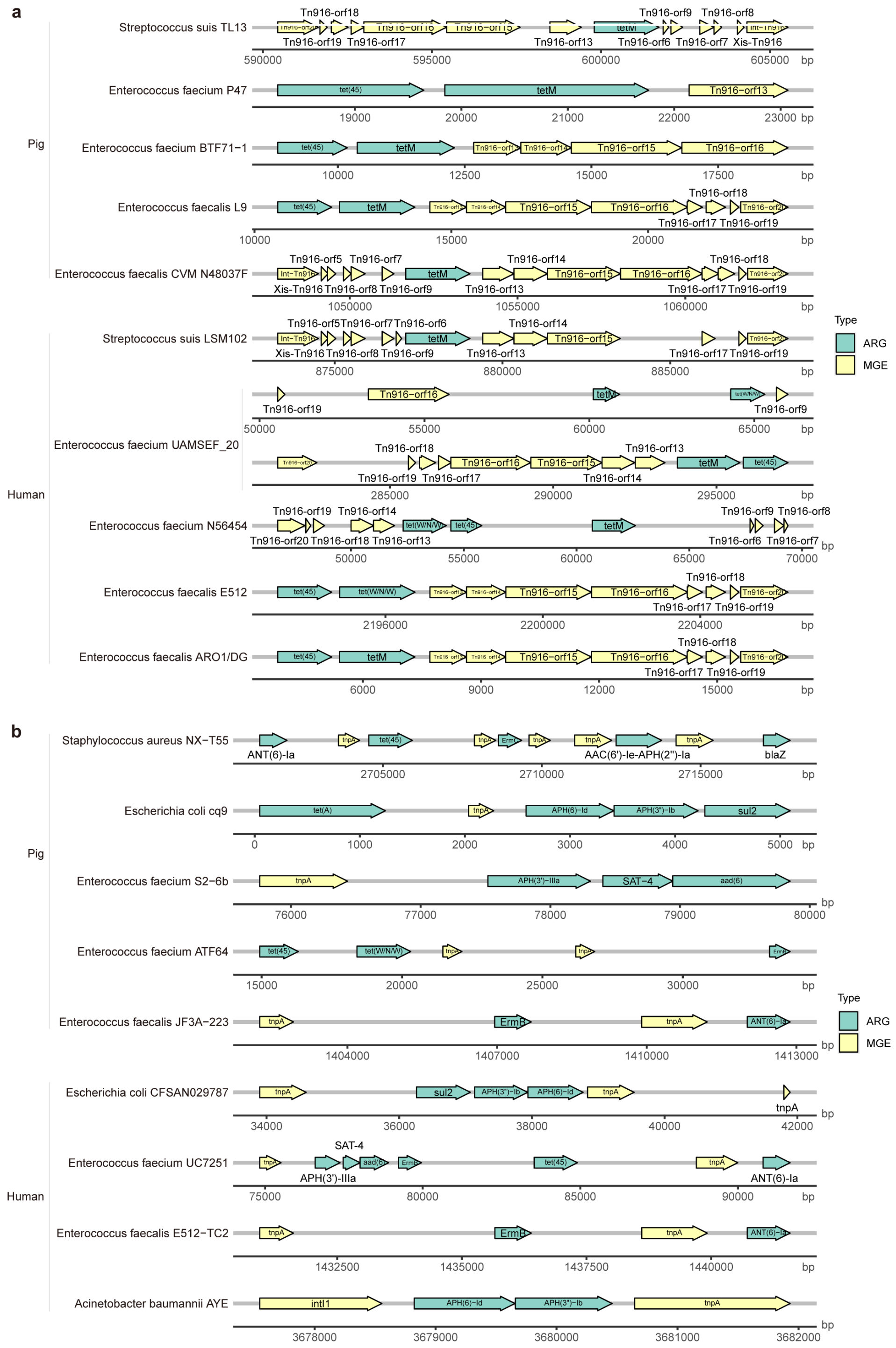

Supplementary Fig. 4. The representative of close linkage relationships between antibiotic resistance genes (ARGs) and mobile genetic elements (MGEs) based on 3,878 genomes of isolated common antibiotic resistant bacteria (ARB)

(a) The representative of close linkages between the Tn916 family and tetracycline ARGs in several common antibiotic resistant bacteria (ARB) from pigs and humans. (b) The representative of close linkages between *tnpA* and various ARGs in several common antibiotic resistant bacteria (ARB) from pigs and humans. Green boxes represent ARGs and the Yellow boxes show MGEs. Source data are provided as a Source Data file.

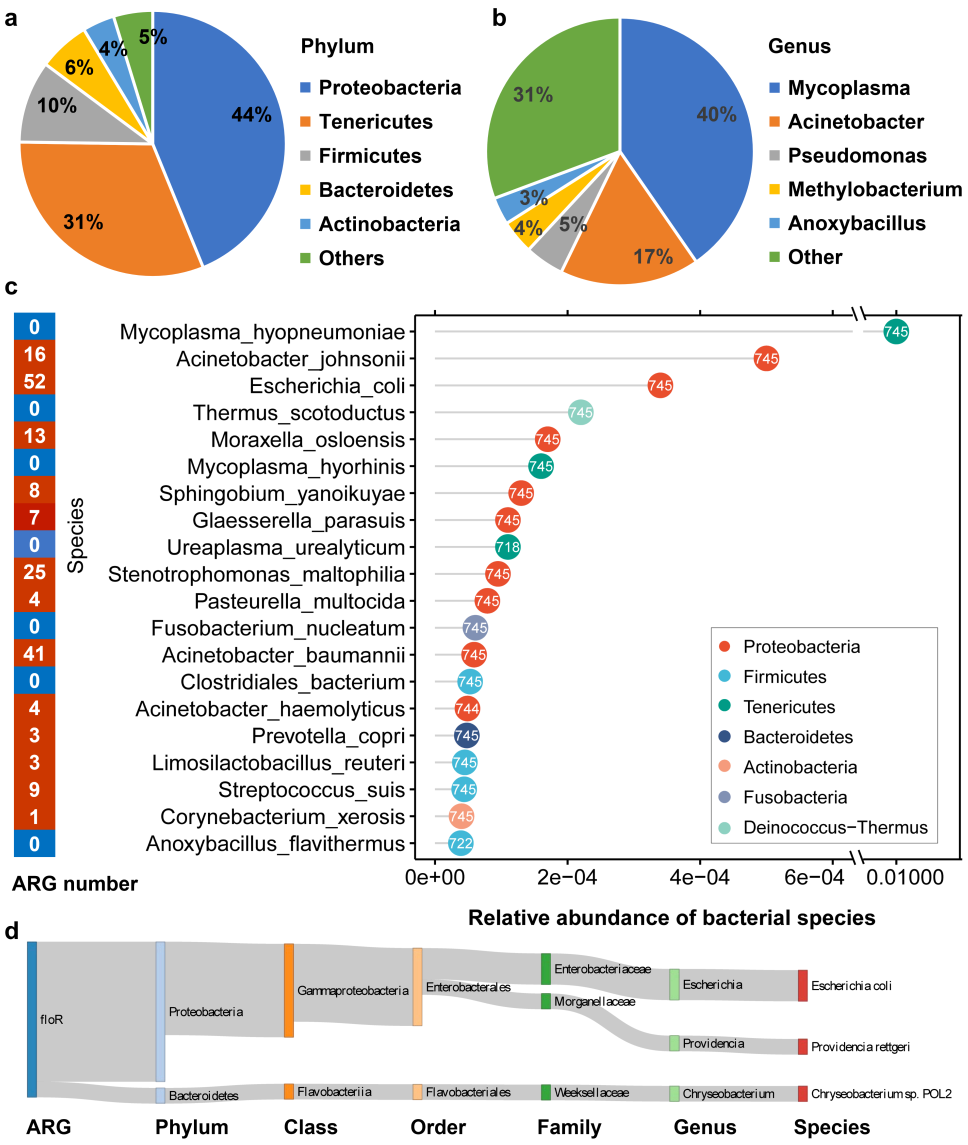

Supplementary Fig. 5. Distribution of host bacteria of ARGs

(a) The compositions of the swine lower respiratory tract microbiome at the phylum level. (b) The compositions of the swine lower respiratory tract microbiome at the genus level. (c) The prevalence and relative abundance of bacterial species whose abundance was listed in the top 20 and the number of ARGs they carried. Colored cycles indicate the phyla that the species belong to. The numbers in circles represent the prevalence of bacterial species in tested samples. The numbers in the left bar show the number of ARGs that bacterial species carried. The *x*-axis indicates the relative abundance of bacterial species. (d) Distribution of host bacteria of the *floR* gene at different taxonomy levels. The colors of the rectangles represent different taxonomy levels (except the first rectangle representing *floR*). The length of the rectangles indicates the number of contigs carrying ARGs. Source data are provided as a Source Data file.

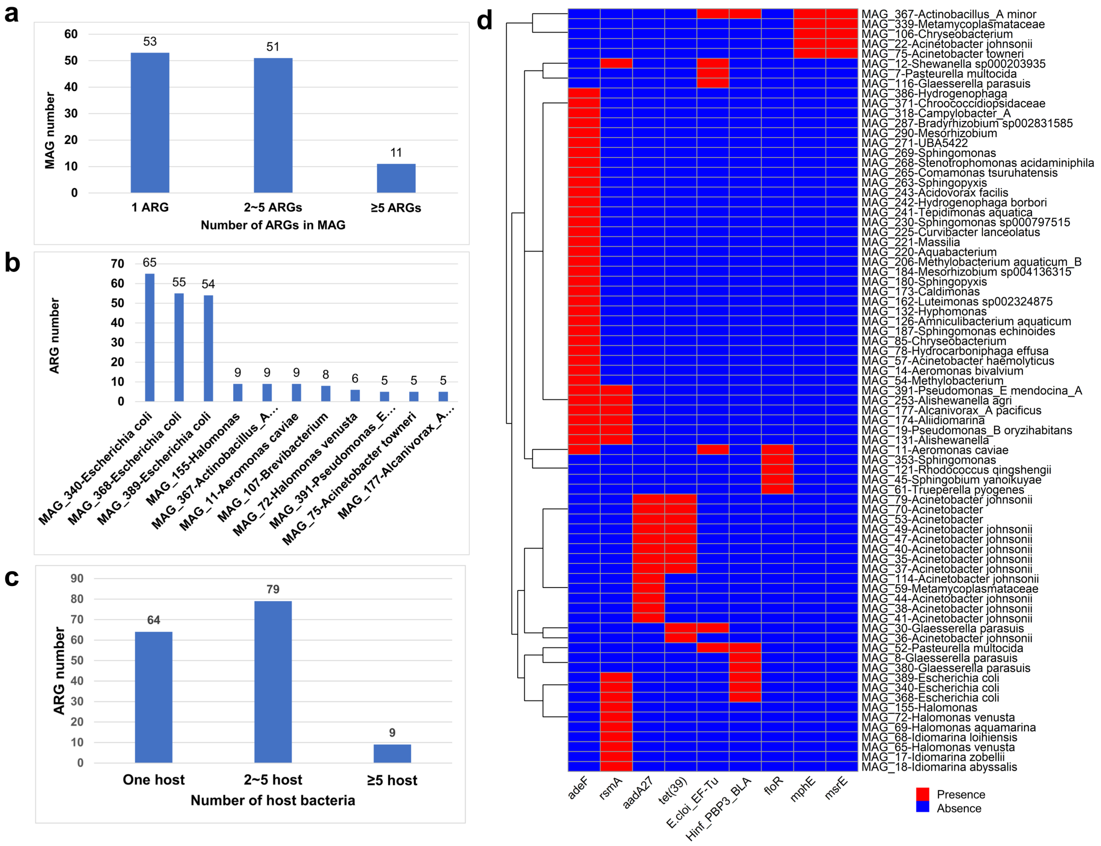

Supplementary Fig. 6. The distribution of ARGs in MAGs

(a) The number of MAGs carrying different numbers of ARGs. (b) The exact number of ARGs carried by 11 MAGs harboring more than five ARGs. (c) The number of ARGs with one or multiple host bacteria. (d) Distribution of nine ARGs with more than five host bacteria in MAGs. Source data are provided as a Source Data file.

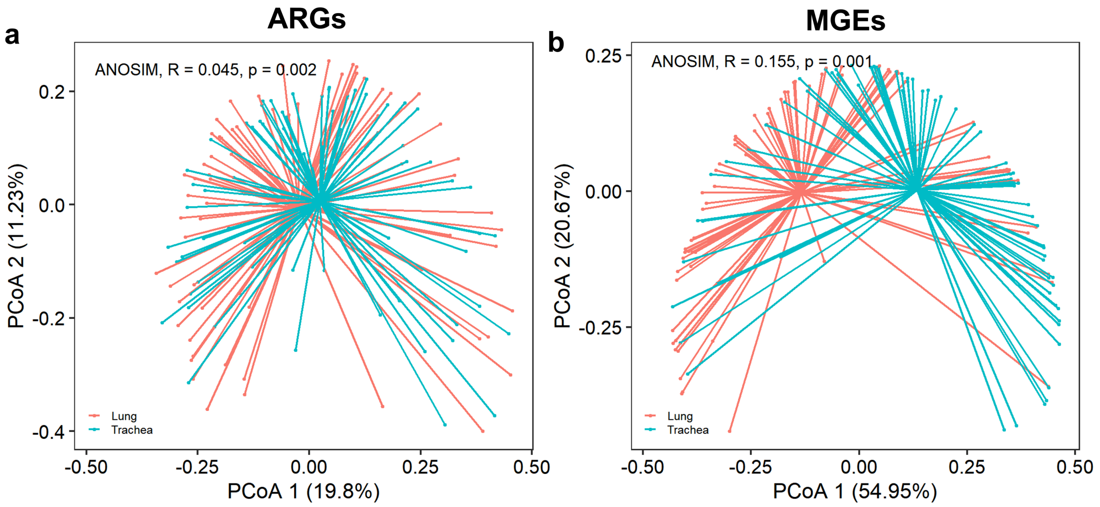

Supplementary Fig. 7. Comparison of the *β*-diversity of antibiotic resistance gene (ARGs) compositions and mobile genetic element (MGEs) compositions between the swine lung and trachea microbiome

(a) Principal coordinates analysis (PCoA) showing the differences in ARG compositions between lung and trachea microbiomes. (b) PCoA showing the differences in MGE compositions between lung and trachea microbiomes. The comparison of the *β*-diversity between lung and trachea microbiomes was performed using analysis of similarity (ANOSIM), and $P < 0.05$ was set as the significance threshold. Source data are provided as a Source Data file.

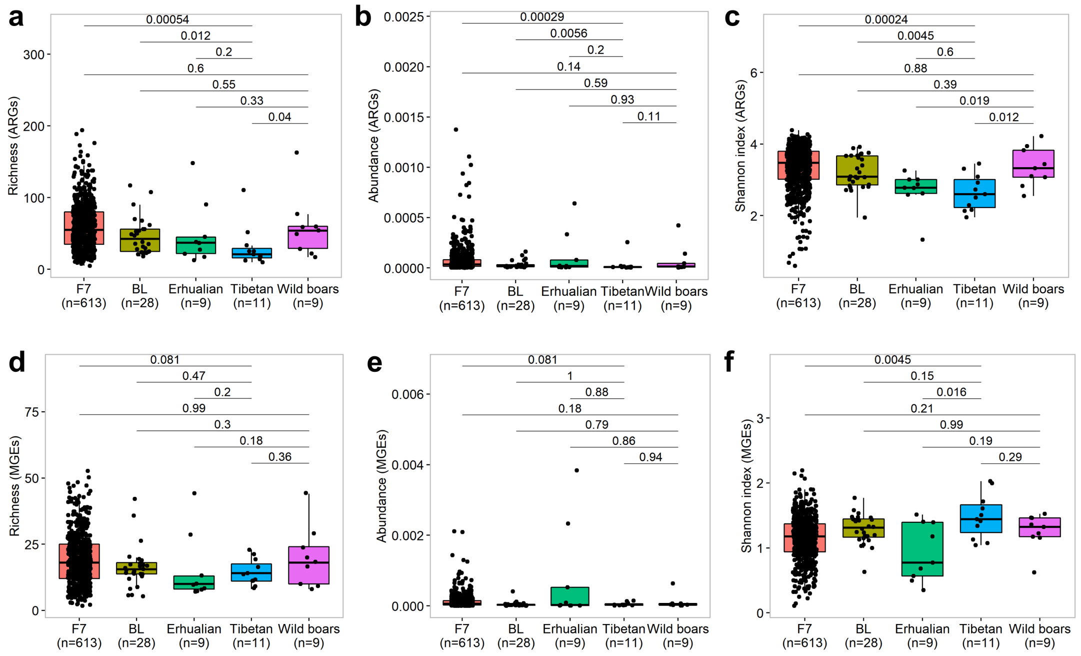

Supplementary Fig. 8. Comparisons of the α-diversity of antibiotic resistance gene (ARG) compositions and mobile genetic element (MGEs) compositions among five different pig populations

(a-c) Comparisons of the richness (number), the total abundance, and the evenness (Shannon index) of ARG compositions among five different pig populations. The *x*-axis shows the α-diversity indices of ARGs, and the *y*-axis indicates pig populations. BL: Berkshire × Licha line. (d-f) Comparisons of the richness (number), the total abundance, and the evenness (Shannon index) of MGE compositions among five different pig populations. All the pairwise comparisons of *α*-diversity of ARG and MGE compositions between each two populations were performed using the two-sided Wilcoxon test. $P < 0.05$ was set as the significance threshold. The *x*-axis shows the α-diversity indices of MGEs, and the *y*-axis indicates pig populations. Boxplots show median, 25th, and 75th percentile. The lower and upper boundaries of whiskers indicate the minima and maxima, respectively. The points laying outside the whiskers of boxplots represent the outliers. Source data are provided as a Source Data file.

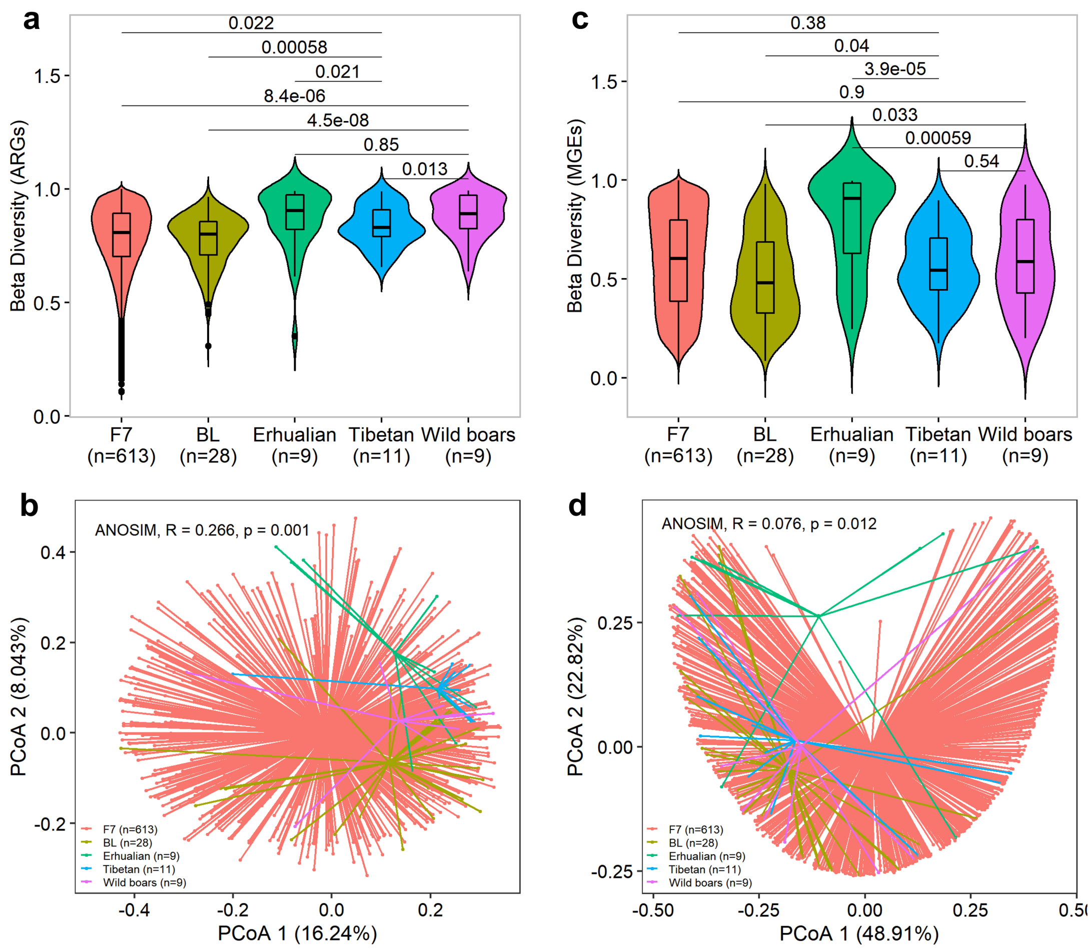

Supplementary Fig. 9. Comparisons of the *β*-diversity of antibiotic resistance gene (ARG) compositions and mobile genetic element (MGE) compositions among five different pig populations

(a) Comparison of the *β*-diversity of the ARG compositions among five different pig populations. The *x*-axis shows the *β*-diversity of ARG compositions, and the *y*-axis indicates pig populations. (b) PCoA showing the differences in the ARG compositions among five different pig populations. (c) The *β*-diversity of the MGE compositions among five different pig populations. The *x*-axis shows the *β*-diversity of MGEs, and the *y*-axis indicates pig populations. (d) PCoA showing the differences in MGE compositions among five different pig populations. The comparisons of the *β*-diversity of ARG and MGE compositions between every two populations were performed using the two-sided Wilcoxon test, and the analysis of similarity (ANOSIM) was used for the comparisons among five pig populations. $P < 0.05$ was set as the significance threshold. Boxplots for (a) and (c) show the median, 25th, and 75th percentile. The lower and upper boundaries of whiskers indicate the minima and maxima, respectively. The points laying outside the whiskers of boxplots represent the outliers. Source data are provided as a Source Data file.

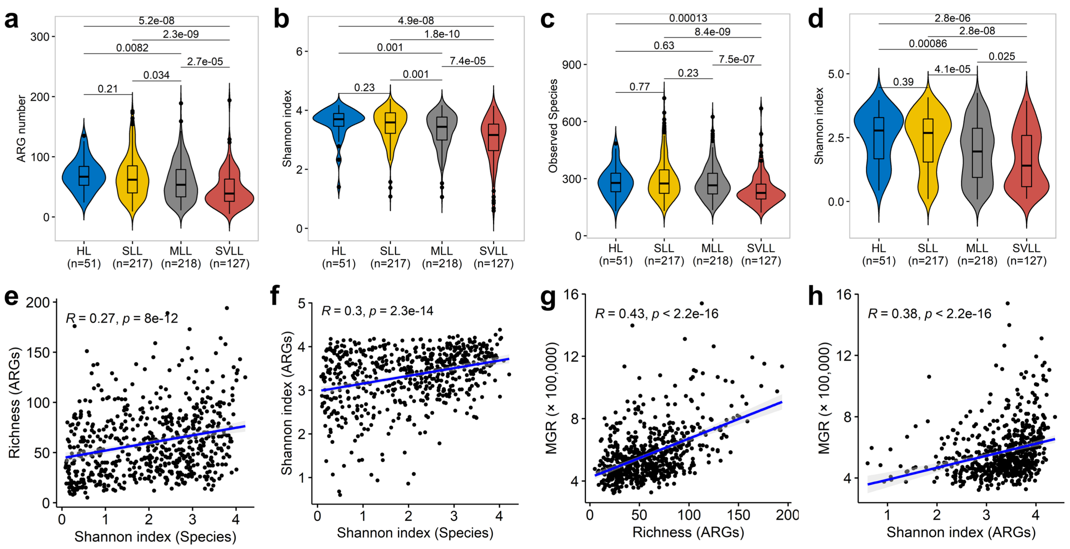

Supplementary Fig. 10. Comparisons of the *α*-diversity of antibiotic resistance gene (ARG) compositions and bacterial compositions among four pig groups with different lung lesions, and the relationship in the *α*-diversity between bacterial and ARG compositions, and between microbial gene richness (MGR) and ARG diversity

(a-d) Comparisons of the richness (number) and the evenness (Shannon index) of ARG and bacterial compositions among four pig groups with different lung lesion levels. The comparisons were performed using the two-sided Wilcoxon test, and $P < 0.05$ was set as the significance threshold. HL: Healthy lung, SLL: slight lung lesions, MLL: moderate lung lesions, and SVLL: severe lung lesions. Boxplots show median, 25th, and 75th percentile. The lower and upper boundaries of whiskers indicate the minima and maxima, respectively. The points laying outside the whiskers of boxplots represent the outliers. (e) The relationship between the evenness (Shannon index) of the microbial composition at the species level and the richness of the ARG composition. (f) The relationship between the Shannon index of microbial composition and ARG composition. (g) The relationship between ARG richness and MGR. (h) The relationship between the Shannon index of ARG composition and MGR. (e-h) The two-sided Spearman's rank correlation in the α-diversity between ARGs and bacterial species, and between ARG and microbial gene richness (MGR) were assessed using the *ggscatter* function in the *ggpubr* R package. Data are presented as the actual value of the corresponding variable obtained for each sample (each point). Source data are provided as a Source Data file.

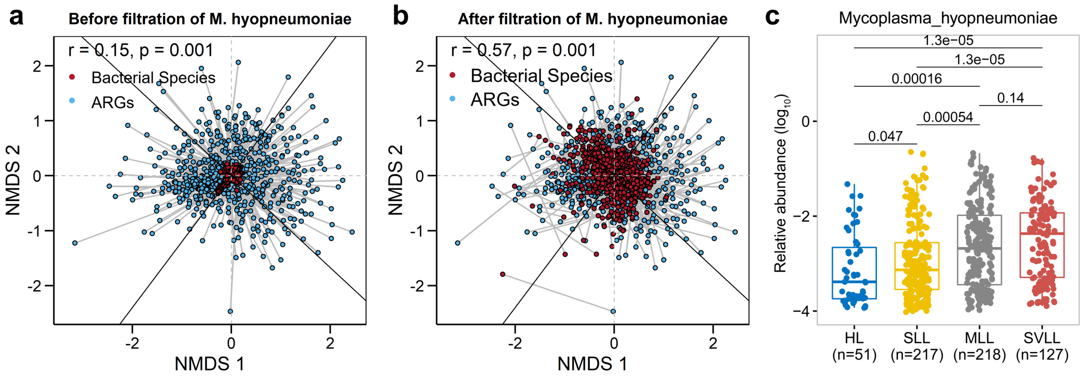

Supplementary Fig. 11. The relationships of *Mycoplasma hyopneumoniae* with the composition of bacterial species and lung lesions

(a-b) Procrustes correlation in the compositions between ARGs and bacterial species before (a) and after (b) filtration of *Mycoplasma hyopneumoniae*. Procrustes correlation analysis was performed by *vegan* R package. (c) Comparison of the abundances of *Mycoplasma hyopneumoniae* among four pig groups with different lung lesion levels. The pairwise comparisons were performed using the two-sided Wilcoxon test. Boxplots show median, 25th, and 75th percentile. The lower and upper boundaries of whiskers indicate the minima and maxima, respectively. The points laying outside the whiskers of boxplots represent the outliers. HL: Healthy lung, SLL: slight lung lesions, MLL: moderate lung lesions, and SVLL: severe lung lesions. Source data are provided as a Source Data file.

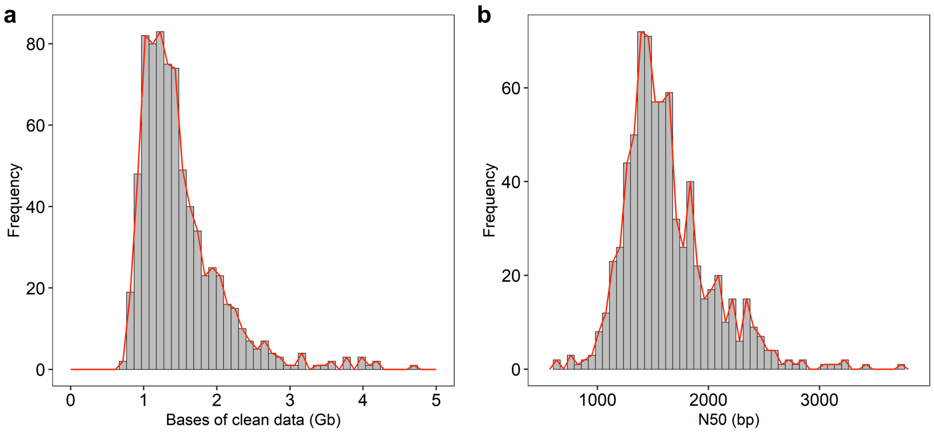

Supplementary Fig. 12. The distribution of data sizes of clean sequences and N50 values of contigs in 745 experimental samples

(a) The distribution of bases of clean sequence data in 745 tested samples. (b) The distribution of N50 values of contigs in 745 tested samples. Source data are provided as a Source Data file.

Supplementary Tables

Supplementary Table 1. The prevalence of the variants of nine clinical ARGs and *mcr* gene in 745 tested samples

Geneid	ARGs	Prevalence
W6920_lung_k141_4434_2	tet(L)	261
5846_lung_k141_963_1	tet(D)	47
5986_lung_k141_5386_1	APH(6)-Id	216
6981_lung_k141_4475_1	sul1	472
ZZE3874_lung_k141_10870_1	sul1	45
6857_lung_k141_14234_2	sul1	115
ZZE4866_lung_k141_10869_1	sul1	7
7105_lung_k141_7958_1	sul1	3
6937_lung_k141_3052_1	sul1	41
All_lung_k141_4361111_1	APH(3")-Ib	64
W7113_lung_k141_24653_1	APH(3")-Ib	95
W7081_lung_k141_14313_1	ANT(6')-Ia	161
W7067_lung_k141_4366_1	ErmB	379
W7093_lung_k141_7088_2	ErmB	1
All_lung_k141_4028562_1	ErmB	14
7111_lung_k141_6308_1	ErmB	35
W7105_lung_k141_17142_1	ErmB	29
All_lung_k141_2875772_1	ErmB	18
All_lung_k141_1808260_2	ErmB	19
All_lung_k141_6477333_1	aad(6)	15
W6914_lung_k141_959_1	ErmT	247
All_lung_k141_1446543_1	MCR-1.2	18
All_lung_k141_109450_2	Mcr-1	29
W6848_lung_k141_10528_1	MCR-3.20	71
W7061_lung_k141_6625_1	MCR-3.4	51
W6860_lung_k141_20230_1	MCR-3.19	11
WB04lung_lung_k141_16296_1	MCR-3.5	7
All_lung_k141_3421855_2	MCR-1.4	12
All_lung_k141_24228_2	MCR-1.6	64
W7083_lung_k141_12952_1	MCR-1.7	9
All_lung_k141_3538161_1	MCR-1.8	21
ZZE3874_lung_k141_4941_1	MCR-4	11
All_lung_k141_4388956_1	MCR-1.3	19

Supplementary Table 2. The distribution of the close linkage relationships (complex) between *tetM* and *Tn916* family in 3,878 genomes of common antibiotic resistant bacteria

**Species**	**Genome number**	***tetM***	***tetM* and *Tn916* complex**	***tetM* and *Tn916* complex (%)**	**From humans**	**From pigs**
Streptococcus suis	94	14	14	15%	3	11
Staphylococcus aureus	976	124	124	13%	118	6
Enterococcus faecalis	168	107	105	63%	96	9
Enterococcus faecium	297	180	179	60%	174	5
Escherichia coli	1172	56	38	3%	14	24
Acinetobacter baumannii	529	2	0	0%	0	0
Pseudomonas aeruginosa	642	0	0	0%	0	0
Total	3878	483	460	12%	405	55

Note: 1. Number of genomes carrying *tetM*; 2. Number of genomes carrying *tetM* and Tn916 complex; 3. Percentage of genomes carrying *tetM* and *Tn916* complex (%); 4. The number of genomes carrying *tetM* and *Tn916* complex from humans; 5. Number of genomes carrying *tetM* and *Tn916* complex from pigs

Supplementary Table 3. The IDs of 23 *Mycoplasma hyopneumoniae* genomes downloaded from the RefSeq database in NCBI

Genome	Origin
GCF_000008205.1_ASM820v1_genomic	RefSeq
GCF_000008225.1_ASM822v1_genomic	RefSeq
GCF_000008405.1_ASM840v1_genomic	RefSeq
GCF_000183185.1_ASM18318v1_genomic	RefSeq
GCF_000400855.1_ASM40085v1_genomic	RefSeq
GCF_000427215.1_ASM42721v1_genomic	RefSeq
GCF_002193015.1_ASM219301v1_genomic	RefSeq
GCF_002213485.1_ASM221348v1_genomic	RefSeq
GCF_004768725.1_ASM476872v1_genomic	RefSeq
GCF_007923985.1_ASM792398v1_genomic	RefSeq
GCF_009831855.1_ASM983185v1_genomic	RefSeq
GCF_009831895.1_ASM983189v1_genomic	RefSeq
GCF_009831905.1_ASM983190v1_genomic	RefSeq
GCF_009831945.1_ASM983194v1_genomic	RefSeq
GCF_009832035.1_ASM983203v1_genomic	RefSeq
GCF_009832075.1_ASM983207v1_genomic	RefSeq
GCF_009832085.1_ASM983208v1_genomic	RefSeq
GCF_009832125.1_ASM983212v1_genomic	RefSeq
GCF_009832175.1_ASM983217v1_genomic	RefSeq
GCF_013402755.1_ASM1340275v1_genomic	RefSeq
GCF_013412725.1_ASM1341272v1_genomic	RefSeq
GCF_021383865.1_ASM2138386v1_genomic	RefSeq
GCF_900660565.1_51334_A01-3_genomic	RefSeq

Supplementary Table 4. Primers used for qPCR

Category	Gene (MAG)	Forword Primer (5'-3')	Reverse Primer (5'-3')
ARG	*tetM*	TGAAAATCCGCACCCTCTAC	AGATTTCCAAAAGGGCATCA
	APH(6)-Id	GATCCTAGACGCATTGCACA	CGTTGCTCCTCTTCTCCATC
	APH(3")-Ib	AGGTAGTTGGCGTCATCGAG	TCACCGTAACCAGCAAATCA
MGE	*Tn916-orf13*	TACAGCGACAGCCAGTGAAC	TCAGCGATACCGTGACTTGA
	Int-Tn916	AGACTGGAGAGAGCCAACGA	AGCCACAAGTTTCCACGAGT
MAG	MAG21	AATGGGGCATGACAATCTTC	TCGGCTTCCTTTATTGCCTA
	MAG26	GGTTAAATCCAACGGGTTCA	TCCCATACGTGCTGCATAAG
	MAG340	AACTTGACGGCGTGTTAAGC	TGGAGCTGCATGACAAAGTC
