# Supplementary material for: Characterization of the pig lower respiratory tract antibiotic resistome
Source: Nat Commun. 2023 Aug 12;14:4868. doi: 10.1038/s41467-023-40587-1 (PMC10423206; doi:10.1038/s41467-023-40587-1)
Supplement: Supplementary file 3 — Description of Additional Supplementary Files [file 41467_2023_40587_MOESM3_ESM.docx]

**Description of Additional Supplementary Files**

**Supplementary Data 1**. Information of experimental pigs and 745 lavage samples of the lower respiratory tract microbiota.

**Supplementary Data 2.** Information of 1,228 ORFs identified as antibiotic resistance protein-coding genes.

**Supplementary Data 3.** The prevalence and average abundance of 372 ARGs in 745 tested samples.

**Supplementary Data 4.** Information of 3,016 ORFs identified as mobile genetic elements.

**Supplementary Data 5.** The information of 3,878 strains of common drug-resistant bacteria isolated from humans or pigs from the Refseq Database.

**Supplementary Data 6.** The information of ARGs harbored in 115 MAGs.

**Supplementary Data 7.** The information of ARGs and MGEs carried by three *Escherichia coli* MAGs.

**Supplementary Data 8.** The prevalence of 372 ARGs in bronchoalveolar lavage fluid and tracheal lavage fluid samples.

Supplementary Data 9. The information of 500 metagenomes of pig gut microbial samples, 46 metagenomes of human lung lavage samples from children with pneumonia, and 118 metagenomes of human lung lavage samples from adult COVID-19 patients.
