# Supplementary material for: Characterization of the pig lower respiratory tract antibiotic resistome
Source: Nat Commun. 2023 Aug 12;14:4868. doi: 10.1038/s41467-023-40587-1 (PMC10423206; doi:10.1038/s41467-023-40587-1)
Supplement: Supplementary file 5 — Reporting Summary [file 41467_2023_40587_MOESM5_ESM.pdf]

nature portfolio

Corresponding author(s): Lusheng Huang, Congying Chen

Last updated by author(s): Jul 26, 2023

Reporting Summary

Nature Portfolio wishes to improve the reproducibility of the work that we publish. This form provides structure for consistency and transparency in reporting. For further information on Nature Portfolio policies, see our Editorial Policies and the Editorial Policy Checklist.

Statistics

For all statistical analyses, confirm that the following items are present in the figure legend, table legend, main text, or Methods section.

n/a	Confirmed	
☐	☒	The exact sample size (*n*) for each experimental group/condition, given as a discrete number and unit of measurement
☐	☒	A statement on whether measurements were taken from distinct samples or whether the same sample was measured repeatedly
☐	☒	The statistical test(s) used AND whether they are one- or two-sided<br>*Only common tests should be described solely by name; describe more complex techniques in the Methods section.*
☐	☒	A description of all covariates tested
☐	☒	A description of any assumptions or corrections, such as tests of normality and adjustment for multiple comparisons
☐	☒	A full description of the statistical parameters including central tendency (e.g. means) or other basic estimates (e.g. regression coefficient) AND variation (e.g. standard deviation) or associated estimates of uncertainty (e.g. confidence intervals)
☐	☒	For null hypothesis testing, the test statistic (e.g. *F*, *t*, *r*) with confidence intervals, effect sizes, degrees of freedom and *P* value noted<br>*Give P values as exact values whenever suitable.*
☒	☐	For Bayesian analysis, information on the choice of priors and Markov chain Monte Carlo settings
☒	☐	For hierarchical and complex designs, identification of the appropriate level for tests and full reporting of outcomes
☐	☒	Estimates of effect sizes (e.g. Cohen's *d*, Pearson's *r*), indicating how they were calculated

Our web collection on statistics for biologists contains articles on many of the points above.

Software and code

Policy information about availability of computer code

Data collection	The metagenomic sequencing data used in this study were sequenced on DNBSEQ-T7 platform (BGI, China).<br>The construction of gene and metagenome assembled genomes (MAGs) catalogs: MEGAHIT (v1.2.9), Prodigal (v2.6.3), CD-HIT (v4.8.1), BWA (v2.2.1), MetaBAT2 (v2.15), Maxbin2 (v2.2.7), CONCOCT (v1.0.0), VAMB (v3.0.2).<br>Taxonomic assignment and abundance calculations of genes and MAGs: BWA (v2.2.1), FeatureCounts (v2.0.1), DIAMOND (v2.0.12.150), BASTA (v1.3.2.3), GTDB-Tk (v1.7.0), Prokka (v1.13), CoverM (v0.6.1).<br>Identification and annotation of ARGs, MGEs, and VFGs: RGI (v 5.2.1), BLASTP (v2.12.0), Kraken2 (v2.1.2), DIAMOND (v0.8.36.98), PHASTER website, Prokka (v1.13), Prodigal (v2.6.3), GTDB-Tk (v1.7.0).<br>qPCR experiment: qPCR was carried out in triplicate with Power SYBR Green Mastermix (Takara, Japan) on an Applied Biosystems 7900 system.
Data analysis	Statistical analysis and visualization: R (v4.1.1), networkD3 (v0.4), vegan (v2.5.7), ggpubr (v0.4.0), pheatmap (v1.0.12), circlize (v0.4.15), ggplot2 (v 3.3.5) and gggenes (v0.4.1) R packages, Gephi (v0.9).<br>All codes produced by this project have been deposited in GitHub repository (https://github.com/zhouyunyan/LungARGs).

For manuscripts utilizing custom algorithms or software that are central to the research but not yet described in published literature, software must be made available to editors and reviewers. We strongly encourage code deposition in a community repository (e.g. GitHub). See the Nature Portfolio guidelines for submitting code & software for further information.

Data

Policy information about availability of data

All manuscripts must include a data availability statement. This statement should provide the following information, where applicable:

- Accession codes, unique identifiers, or web links for publicly available datasets
- A description of any restrictions on data availability
- For clinical datasets or third party data, please ensure that the statement adheres to our policy

The metagenomic sequencing data have been deposited in the Genome Sequence Archive (GSA) repository under accession ID: CRA007668 (https://ngdc.cncb.ac.cn/gsa/browse/CRA007668). MAGs and microbial gene catalog used in this study are available in the GSA database under accession codes: GWHBPMO00000000~ GWHBQBU00000000 and OMIX002571 (https://ngdc.cncb.ac.cn/bioproject/browse/PRJCA010893). Raw data related to figures were showed in Supplementary Tables, Supplementary Data or Source data. Source data and codes producing Figures are also deposited in the GitHub repository (https://github.com/zhouyunyan/LungARGs) .

Databases used in this study: Uniprot TrEMBL, CARD (v 3.1.4), SARG (v 2.2).
Twenty-three genomic sequences of Mycoplasma hyopneumoniae were downloaded from the NCBI RefSeq database. 3,878 bacterial genomes were downloaded from the NCBI RefSeq database. 6,339 MAGs of pig gut metagenomes from our previous study are available in China National GeneBank DataBase with accession code CNP0000824 (https://db.cngb.org/search/?q=CNP0000824). Forty-six and 118 metagenomic sequencing data of BAL fluid samples were downloaded from GenBank repository (accession number SRP119571, https://www.ncbi.nlm.nih.gov/sra/?term=SRP119571) and NCBI Sequence Read Archive under project numbers PRJNA687506 (https://www.ncbi.nlm.nih.gov/bioproject/?term=PRJNA687506), respectively.
These databases and datasets are public.

Human research participants

Policy information about studies involving human research participants and Sex and Gender in Research.

Reporting on sex and gender	*Use the terms sex (biological attribute) and gender (shaped by social and cultural circumstances) carefully in order to avoid confusing both terms. Indicate if findings apply to only one sex or gender; describe whether sex and gender were considered in study design whether sex and/or gender was determined based on self-reporting or assigned and methods used. Provide in the source data disaggregated sex and gender data where this information has been collected, and consent has been obtained for sharing of individual-level data; provide overall numbers in this Reporting Summary. Please state if this information has not been collected. Report sex- and gender-based analyses where performed, justify reasons for lack of sex- and gender-based analysis.*
Population characteristics	*Describe the covariate-relevant population characteristics of the human research participants (e.g. age, genotypic information, past and current diagnosis and treatment categories). If you filled out the behavioural & social sciences study design questions and have nothing to add here, write "See above."*
Recruitment	*Describe how participants were recruited. Outline any potential self-selection bias or other biases that may be present and how these are likely to impact results.*
Ethics oversight	*Identify the organization(s) that approved the study protocol.*

Note that full information on the approval of the study protocol must also be provided in the manuscript.

Field-specific reporting

Please select the one below that is the best fit for your research. If you are not sure, read the appropriate sections before making your selection.

☒ Life sciences ☐ Behavioural & social sciences ☐ Ecological, evolutionary & environmental sciences

For a reference copy of the document with all sections, see nature.com/documents/nr-reporting-summary-flat.pdf

Life sciences study design

All studies must disclose on these points even when the disclosure is negative.

Sample size	To characterize the profiles of the pig lower respiratory tract antibiotic resistome, we collected pig lower respiratory tract microbial samples as many as possible. A total of 745 lower respiratory tract microbial samples including 670 bronchoalveolar lavage (BAL) fluid samples, 74 tracheal lavage fluid samples, and one esophageal lavage fluid sample from 675 experimental pigs were used in this study.
Data exclusions	All 745 samples were used to characterize the pig lower respiratory tract antibiotic resistome. No data was excluded.
Replication	A total of 745 pig lower respiratory tract microbial samples from 675 experimental pigs were used in this study . These experimental pigs were from five populations. Sample replication was not relevant for characterization of the pig lower respiratory tract antibiotic resistome because this is a observation study. However, to validate the abundance changes of ARGs, MGEs and MAGs, and the co-abundance relationships

	between them, we performed the quantitative PCR (qPCR). A total of 23 samples containing both the samples detected and undetected the abundances of these ARGs, MGEs and MAGs in the metgenomic sequencing analysis were used for qPCR analysis. qPCR was carried out in triplicate with Power SYBR Green Mastermix (Takara, Japan) on an Applied Biosystems 7900 system. The excellent replication result was obtained.
Randomization	Randomization was not relevant to this study and was not employed. All 745 samples from 675 experimental pigs were used for metagenomic sequencing and for determining the characterization of the pig lower respiratory tract antibiotic resistome. We controlled for potential sources of confounding by (a) animals were reared in standardized housing and feeding conditions. (b) collection method of samples was unified.
Blinding	Blinding is not relevant to this study and was not employed because this is an observational study.

Reporting for specific materials, systems and methods

We require information from authors about some types of materials, experimental systems and methods used in many studies. Here, indicate whether each material, system or method listed is relevant to your study. If you are not sure if a list item applies to your research, read the appropriate section before selecting a response.

Materials & experimental systems

n/a	Involved in the study
☒	☐ Antibodies
☒	☐ Eukaryotic cell lines
☒	☐ Palaeontology and archaeology
☐	☒ Animals and other organisms
☒	☐ Clinical data
☒	☐ Dual use research of concern

Methods

n/a	Involved in the study
☒	☐ ChIP-seq
☒	☐ Flow cytometry
☒	☐ MRI-based neuroimaging

Animals and other research organisms

Policy information about studies involving animals; ARRIVE guidelines recommended for reporting animal research, and Sex and Gender in Research

Laboratory animals	The experimental pigs were from five populations: F7 pigs of a mosaic population (n = 618), Erhualian pigs raised on the Changzhou farm (n = 9), Berkshire × Licha line pigs from the Dingnan farm (n = 28), wild boars (n = 9), and Tibetan pigs from the Linzhi farm (n =11). The age of F7 pigs, Erhualian pigs and Berkshire × Licha line pigs were 240 days , 60 days, and 195~265 days , respectively. Wild boars and Tibetan pigs are adult pigs, but the exact ages were unknown. All F7 pigs were housed in a uniformed farm of Jiangxi Agricultural University in Nanchang and provided commercial formula feed containing 16% crude protein and 3100 kcal/kg digestible energy and 0.78% lysine. All Erhualian, Tibetan, and Berkshire × Licha pigs were fed with commercial formula feed satisfying the standard pig nutritional requirements. Water was provided ad libitum from nipple drinkers. All experimental pigs were slaughtered for sample collection by bleeding after electrical stunning.
Wild animals	A total of nine wild boars were used in this study. To collect bronchoalveolar lavage fluid samples, the animals were anesthetized and transported to the laboratory. All nine wild boas were slaughtered by bleeding after electrical stunning, and disposed properly after sampling. We didn't known the exact age of these wild boars, but all nine animals were adult wild boars. The project was supported by the government.
Reporting on sex	A total of 675 pigs were used in this study, including 371 males and 304 females. In more details, F7 pigs of a mosaic population (n = 618, 264 female and 354 male), Erhualian pigs raised in the Changzhou farm (n = 9, 4 female and 5 male), Berkshire × Licha line pigs from the Dingnan farm (n = 28, 22 female and 6 male), wild boars (n = 9, 3 female and 6 male), and Tibetan pigs from the Linzhi farm (n = 11, all female).
Field-collected samples	All lavage samples were obtained by rinsing bronchoalveoli or trachea with sterile phosphate-buffered saline (PBS) immediately after slaughter. A total of 670 bronchoalveolar lavage (BAL) fluid samples were collected from five pig populations. Seventy-four tracheal lavage fluid samples and one esophageal lavage fluid sample were obtained from F7 pigs of the mosaic pig population. All experimental pigs were sampled under standard procedures within 30 min after slaughter. The house was under natural temperature and photoperiod.
Ethics oversight	All procedures involved in experimental pigs were conducted according to the guidelines for the care and use of experimental animals established by the Ministry of Agriculture and Rural Affairs of China. The project was also approved by Animal Care and Use Committee (ACUC) in Jiangxi Agricultural University (No. JXAU2011-006).

Note that full information on the approval of the study protocol must also be provided in the manuscript.
